# Venular-centered thrombo-inflammation drives microvascular failure after arterial recanalization in acute mesenteric ischemia

**DOI:** 10.1038/s43856-026-01878-y

**Published:** 2026-08-28

**Authors:** Déborah François, Lambert Kernanet, Melinda Bajul, Abigail Brami, Véronique Arocas, Marie-Christine Bouton, Dominique Cazals-Hatem, Kevin Guedj, Benoit Ho-Tin-Noé, Olivier Corcos, Yacine Boulaftali, Alexandre Nuzzo

**Affiliations:** 1 Laboratory for Vascular Translational Science, INSERM 1148, Université Paris Cité, Paris, France; 2 Department of Radiology, AP-HP, Beaujon Hospital, Clichy, France; 3 Department of Histopathology, AP-HP, Beaujon Hospital, Clichy, France; 4 Center for Intestinal Failure, Intestinal Stroke Center, Reference Centre for Rare Disease MarDI, Department of Gastroenterology, IBD, and Nutritional Support, AP-HP, Beaujon Hospital, Clichy, France; 5 Optimisation Thérapeutique en Neuropsychopharmacologie, Inserm UMR-S 1144, Faculté de Pharmacie, Université Paris Cité, Paris, France

**Keywords:** Gastrointestinal models, Gastrointestinal diseases

## Abstract

**Background:**

Acute mesenteric ischemia (AMI) remains associated with high mortality despite prompt revascularization, suggesting that downstream ischemia–reperfusion injury contributes to poor outcomes. However, the microvascular mechanisms underlying this process are not entirely defined.

**Methods:**

We analyzed admission blood samples from patients with arterial AMI and non-ischemic controls enrolled in the SURVIBIO diagnostic study (NCT03518099). We also investigated thrombo-inflammatory responses in a murine superior mesenteric artery occlusion (SMAO) model. In mice, intravital microscopy was used to directly visualize mesenteric microcirculatory flow and thrombo-inflammatory events during ischemia–reperfusion.

**Results:**

Patients with AMI display a marked systemic thrombo-inflammatory profile, characterized by elevated inflammatory markers, neutrophil activation, platelet activation, and alterations in coagulation-related proteins, which are closely mirrored in the SMAO model. Intravital microscopy reveals a dissociation between arterial and microvascular reperfusion: while arteriolar flow partially recovers after recanalization, venular perfusion remains severely impaired and is associated with early blood cell stasis and stable venular thrombus formation. Thrombi develop through a sequential process initiated during ischemia and amplified during reperfusion.

**Conclusion:**

Together, these findings suggest that venular-centered thrombo-inflammation is closely associated with microvascular dysfunction and intestinal injury in arterial AMI. This work provides a framework for targeting thrombo-inflammatory pathways beyond arterial reperfusion that may extend to other clinical forms of AMI, including non-occlusive mesenteric ischemia (NOMI).

## Plain Language Summary

When blood supply to the intestines is suddenly blocked, it causes a life-threatening condition called acute mesenteric ischemia. Even when doctors clear the main artery blockage, many patients still die. This study investigated why the gut fails to recover. By analyzing human blood samples and viewing mouse intestines under special microscopes, researchers found that the issue lies in the smallest blood vessels. While larger arteries cleared up, the tiny veins remained blocked by inflammation and small blood clots that formed during the blockage and grew worse afterward. This shows that simply opening the main artery is not enough. Future treatments must also target this microscopic clotting and inflammation to successfully save lives and protect the intestines.

## Abstract (web summary)

François et al. investigate the mechanisms underlying microvascular failure following arterial recanalization in acute mesenteric ischemia. Findings reveal that early venular thrombosis and localized inflammation compromise microcirculatory recovery, highlighting potential therapeutic targets to preserve gut viability.

## Introduction

Acute mesenteric ischemia (AMI) is a life-threatening gastrointestinal emergency that remains associated with a mortality rate of 30–50%, despite advances in diagnosis, arterial revascularization techniques, and the development of specialized referral centers^1–3^. Prompt restoration of arterial blood flow is essential to prevent transmural intestinal necrosis and its systemic complications, including sepsis and multiorgan failure. However, clinical outcomes remain poor even after technically successful revascularization, indicating that downstream mechanisms beyond large-vessel recanalization critically contribute to intestinal injury and prognosis.

Ischemia-reperfusion (IR) injury is increasingly recognized as a central driver of tissue damage and remote multiorgan failure in AMI. Although reperfusion is required to salvage ischemic bowel, the abrupt restoration of blood flow paradoxically exacerbates injury through oxidative stress, endothelial dysfunction, and immune activation. In the intestine, IR compromises epithelial integrity and barrier function, facilitating bacterial translocation and triggering systemic inflammatory responses that may rapidly progress to organ failure^4^. Despite its clinical relevance, no targeted therapies currently exist to mitigate IR in AMI. Management remains largely focused on restoring vascular patency, with no interventions aimed at enhancing intestinal tolerance to ischemia or promoting recovery after reperfusion.

Experimental models of superior mesenteric artery occlusion (SMAO) have shown that inflammation is an early and sustained feature of both ischemia and reperfusion^5,6^. Hypoxia-induced oxidative stress during ischemia is amplified upon reperfusion by oxygen influx and activation of endothelial cells, neutrophils, macrophages, and platelets, resulting in profound microvascular dysfunction^7^. Growing evidence indicates that inflammatory and thrombotic pathways are tightly interconnected in this setting, forming a thrombo-inflammatory response that amplifies tissue injury. Endothelial cell-mediated T-cell-dependent neutrophil recruitment^8^, excessive reactive oxygen species (ROS) production^9^, neutrophil extracellular traps (NETs) formation^10,11^, and platelet–fibrin deposition^12–16^, have all been implicated in intestinal IR injury, yet these processes are often studied separately. Mechanistic insights into the role of P-selectin in intestinal ischemia have largely emerged from experimental IR models. In transient ischemia, P-selectin primarily mediates leukocyte rolling and adhesion at the endothelial surface^17^. In contrast, models of prolonged complete mesenteric artery occlusion have revealed a broader role for P-selectin in orchestrating thrombo-inflammatory injury by promoting platelet–leukocyte interactions, inflammatory cell infiltration, cytokine production, and subsequent tissue damage^18–20^. Accordingly, blockade or genetic deletion of P-selectin markedly reduces intestinal injury and improves survival in these severe IR models.

In AMI, IR disrupts the intestinal barrier, allowing bacterial translocation that activates Toll-like receptor (TLR) signaling and further amplifies inflammation and tissue injury^21,22^. These reciprocal interactions between thrombosis and inflammation underscore AMI as a thrombo-inflammatory disease. However, the mechanisms driving intestinal microvascular thrombo-inflammation during AMI remain poorly understood.

To address this gap, we combined a translational analysis of thrombo-inflammatory plasma biomarkers from patients with AMI enrolled in the SURVIBIO biobank with mechanistic studies in a standardized mouse model of SMAO. Using intravital microscopy, we performed a kinetic analysis of mesenteric arterioles and venules during IR, providing real-time insight into the development of intestinal microvascular thrombo-inflammation. Together, these complementary clinical and experimental approaches provide new mechanistic insight into the thrombo-inflammatory pathways underlying AMI and identify potential therapeutic targets beyond arterial recanalization.

## Methods

### Human plasma collection

Patients were prospectively enrolled in the SURVIBIO diagnostic study (NCT03518099, registration date: 2018-11-16), a cross-sectional study including patients with AMI and controls presenting with non-ischemic acute abdominal pain, as previously described^23^. All participants consent to participate in the SURVIBIO study. Patients with AMI were managed at the intestinal stroke center (SURVI, Beaujon Hospital, Clichy, France), a tertiary referral center providing 24/7 standardized multidisciplinary care^24^. Standard treatment included aspirin, heparin, antibiotics, and urgent endovascular or surgical revascularization^24,25^. In patients with suspected transmural necrosis, laparotomy was performed to assess bowel viability and guide intestinal resection^26^. Serum samples from 20 patients (10 with arterial AMI and 10 non-AMI controls) included between January 2016 and March 2018, were selected for analysis. Baseline demographic, clinical, and biological data were collected at admission. Blood samples were obtained on admission, immediately centrifuged, and serum aliquots were stored at −80 °C for biomarker analyses. Within an untargeted proteomics discovery workflow, we specifically examined thrombo-inflammatory markers, including P-selectin and myeloperoxidase (MPO). Detailed proteomics methods are provided in the Supplementary Method. Serum levels of endotoxin were also assessed (endotoxin chromogenic quantification kit, ThermoFischer). The study was approved by the Ethics Committee of Paris-Nord Val de Seine University Hospitals, and all participants provided written informed consent prior to enrollment.

### Superior mesenteric artery occlusion (SMAO) mouse model

Before surgery, all mice received a s.c. injection of analgesia (1 mg/kg buprenorphine) at least 30 min before anesthesia. After the anesthesia induction at 4%, anesthesia maintenance was achieved by 1.5% isoflurane via facemask during SMAO (2 h). Temperature homeostasis was achieved through the use of a thermostated heating plate. Using aseptic technique, a midline laparotomy was performed, the intestines were eviscerated, and the mesentery exposed. The superior mesenteric artery was identified and clamped using an atraumatic microvascular clamp. Following 60 min of intestinal occlusion, the atraumatic clamp was removed. During the occlusion (1 h) and recanalization (1 h) phases, the intestine and mesentery were constantly hydrated with sterile NaCl 0.9%. Following surgery, animals were euthanized by cervical dislocation. A group size of 8–15 animals per group, sham (laparotomy without vascular occlusion) or SMAO mice, was determined to optimize the animal model, taking into account the statistical risk of error (5%) and the power of the tests (80%). Forty-five animals were used in total. No animals or prior experimental criteria were excluded during the study. Following the sacrifice, specific post-mortem data points were excluded from analysis due to technical artifacts during sample execution. Live procedures were non-blinded, but all mice were assigned random identification numbers so samples were fully anonymized prior to analysis. Post-sacrifice data processing and image analysis were conducted by two independent observers blinded to treatment groups. To control for potential confounders, the sample measurement order was randomized based on animal numbers. Primary outcome measures included the presence or absence of venular thrombosis.

All procedures adhered to the ethical guidelines of the European Directive (2010/63/EU) and were approved by the ethical committee of the French Ministry of Higher Education and Research. The experiments followed the Animal Research: Reporting In Vivo Experiments 2.0 guidelines and were approved under the reference numbers n°40873 & 46125. All animals were maintained under standard conditions and kept on a 12-h light/dark cycle with food and water given ad libitum (maximum five animals per cage). Eight- to 12-week-old male and female C57BL/6J mice (Janvier Laboratories) were acclimated for at least 1 week before experimentation.

### Real-time intravital imaging

Thrombus formation was visualized in the mesenteric microcirculation, specifically in paired distal arterioles (50–100 μm) and venules (100–250 μm) arising from the superior mesenteric artery, rather than in intestinal capillaries, using a fluorescence macroscope. (Z16 APO, Leica Microsystems) equipped with a thermostated heating plate and an objective connected to a CMOS camera (ORCA-Flash 4.0 LTPlus; Hamamatsu Photonics). Data acquisition and analysis were done using Micromanager (Open Source Software). All fluorescent markers were administrated intravenously into the retroorbital sinus. Rhodamine 6G was used to label platelets and leukocytes, AlexaFluor488 (Invitrogen)-conjugated rat anti-mouse Ly6G (4 µg; BioXCell; BE0075-1; Clone 1A8; lot number #93002301) was used to stain neutrophils, AlexaFluor555 (Invitrogen)-conjugated rat anti-mouse GPIX (4 µg; Emfret; M051-0; Clone Xia.B4; lot number #0510-C) was used to stain platelets and AlexaFluor647 (Invitrogen)- conjugated mouse anti-mouse anti-fibrin (4 µg; Sigma; MABS2155; Clone 59D8) was used to stain fibrin. To monitor cell recruitment kinetics during ischemia and recanalization, 10 s intravital imaging acquisitions were performed before SMAO, at T0, T20, T40, and T60  min after SMAO and at T0, T20, T40, and T60 min after recanalization. For all timepoints, leukocytes number per field of view were counted in the arterioles (10-s acquisition time), and a global fluorescence was measured in the venules (three measures per acquisition). Individual fluorescent cells were tracked in arterioles. In venules, where blood stasis and thrombus formation precluded individual cell tracking, fluorescence intensity was quantified within predefined regions of interest over the 2-h imaging period as a surrogate marker of microvascular congestion. Venular thrombosis occurrence was assessed in each group and is expressed as a percentage of mice developing venular thrombosis during recanalization after SMAO.

### Laser Doppler interferometry

Intestinal mesenteric perfusion was analyzed through blood cell velocity in micro-vessels measured using a single-point laser Doppler vibrometer with an integrated CCD video camera (Polytec). To allow specific measurement of blood cell velocity, breathing-related and flow-induced vessel wall vibrations were identified by their bidirectional associated signal and eliminated by applying a low-pass filter set at 2 kHz. Frequencies due to the unidirectional out-of-plane vibrations caused by circulating blood cells were converted to speed according to the formula *v* = (Δ*f* × *λ*)/(2 × cos*α*), where *v* is the blood cell velocity, Δ*f* is the Doppler-frequency shift, *λ* is the wavelength of the emitted wave (633 nm), and *α* is the angle between the blood cell direction and the incident laser beam, which was estimated at 80°. Data were recorded and treated using the Polytec Vibrometer Software. Perfusion images were acquired at baseline (before SMAO), at T0, T20, T40, and T60 min after SMAO and at T0, T20, T40, and T60  min after recanalization. Perfusion data were expressed as a percentage of baseline.

### Mice histological staining, scoring, and intestinal damages quantification

Following euthanasia of mice groups, segments of intestine were harvested, fixed in 4% paraformaldehyde and embedded in Paraffin for histological analysis. Intestinal sections of 5 µm thickness were stained with hematoxylin and eosin and subjected to histological scoring to evaluate tissue damage. Mucosal damage score was calculated as described by Chiu et al.^27^. Macroscopic assessment of intestinal damaged sections was performed by measuring damaged sections along the entire length of the extracted intestine and is expressed as a percentage of total intestinal length. The Chiu score and quantification of intestinal damages was assessed blindly by two pathologists.

### Immunofluorescence staining

For immunofluorescent staining, paraformaldehyde-fixed paraffin sections of intestine and thrombotic mesenteric vessels were subjected to rehydration and antigen retrieval. After blocking with PBS/BSA 3% for an hour, the sections were incubated overnight with primary antibodies. The following day, sections were washed and incubated with secondary antibodies for 2 h. Stained sections were mounted using Fluoromount containing DAPI (Abcam). The following antibodies were used: Rabbit anti-mouse CD42b (1:100; Abcam; ab183345; Clone SP219; Lot number #1031863-36), Rat anti-mouse CD45 (1:1000; Biolegend; 103102; Clone 30F11; Lot number #B204309), Rabbit anti-mouse Fibrin/Fibrinogen (1:100; Dako; A0080; Lot number #41868236), Rat anti-mouse LY6G (1:50; BD pharmingen; 551459; Clone 1A8; Lot number #3193508), and Rabbit anti-mouse Neutrophil elastase (1:50; Abcam; ab314916; Clone RM1077; Lot number #1140342-12). Secondary antibodies were Goat anti-rabbit 647 (1:800; Invitrogen, a31573) Goat anti-rat Cy3 (1:800; Invitrogen; a10522), Goat anti-mouse 488 (1:800; Invitrogen; a110329).

### Protein dosages

Plasma samples from sham or SMAO mice group were prepared and used to assess levels of interleukine-6 (IL-6) (Invitrogen), myeloperoxidase (MPO; R&D System), soluble P-selectine (sP-sel, R&D System), thrombin/anti-thrombin (TAT) complex (AssayMax) and endotoxin (Thermo Fischer) according to the manufacturer’s specifications.

### Statistical analysis

Data are presented as means ± SEM. SMAO mice data were compared to Sham mice data using a nonparametric Mann–Whitney statistic test. Values of *p* < 0.05 were considered statistically significant. For patient’s characteristics, continuous variables were presented as median (IQR). Variables were compared using the Mann–Whitney test. All tests were two-sided, and p ≤ 0.05 was considered significant.

### Ethical approval

The clinical trial was conducted in accordance with the principles of the Declaration of Helsinki and Good Clinical Practice (GCP) guidelines. Ethical approval was obtained, and the trial is reference as NCT03518099 (Registration date: 2018-11-16). Ethical approval for the clinical cohorts was obtained from the French institutional ethics committees (Comité de Protection des Personnes/CPP, Assistance Publique – Hôpitaux de Paris). SURVIBIO protocol was approved by CPP (17 09 09) Sud Méditerranée IV, and SURVI Cohort protocol was approved by CPP (20.03.11.55523) Sud Méditerranée V. No additional IRB approval was required as the current analysis falls entirely within the scope and ethics coverage of these existing approvals. All participants provided written informed consent prior to enrollment. Participants were informed about the study objectives, procedures, potential risks and benefits, and their right to withdraw at any time without consequences. Participant confidentiality was maintained through anonymization of data, and all study records were securely stored.

All animal procedures were conducted in accordance with national and institutional guidelines for the care and use of laboratory animals. The study protocol was approved by the Animal Ethics Committee of the French Ministry of Higher Education and Research. The experiments followed the Animal Research: Reporting In Vivo Experiments 2.0 guidelines and were approved under the reference numbers n°40873 & 46125. Efforts were made to minimize animal suffering and to reduce the number of animals used while maintaining scientific validity.

## Results

### Thrombo-inflammation biomarkers in patients and mice

**Fig. 1:**
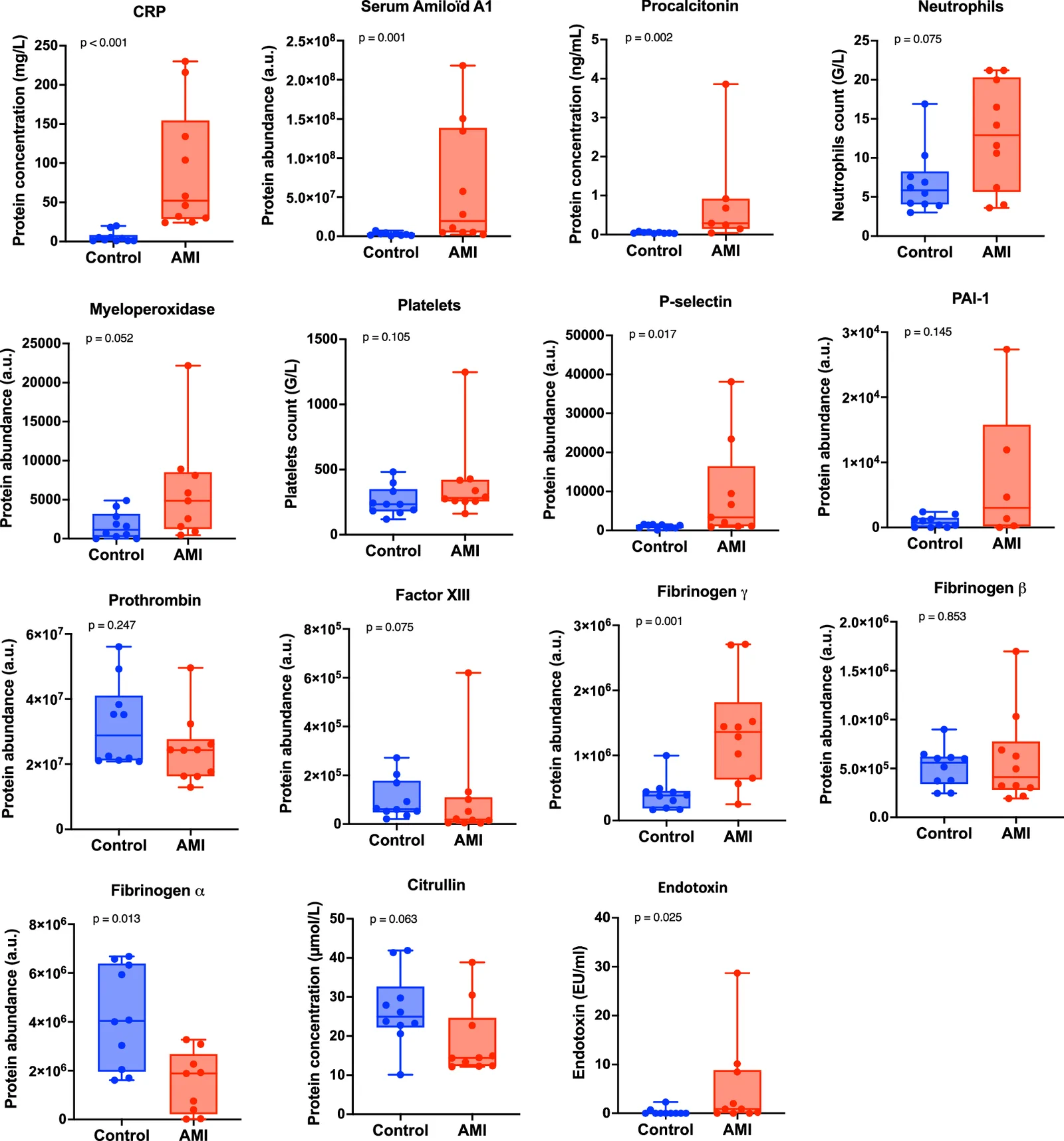
Biomarkers of thrombo-inflammation and epithelial damage in AMI patients. Biomarkers of thrombo-inflammation and epithelial damage in AMI patients (*n* = 10) and control patients (*n* = 10). Proteomics data are expressed in arbitrary units (a.u). Quantitative variables are expressed as median and IQR. Variables were compared using the Mann–Whitney test. All tests are two-sided. *p* ≤ 0.05 was considered significant. *p*-value is indicated on each graph. CRP: C-reactive protein. AMI: acute mesenteric ischemia.

We analyzed admission blood samples from 20 patients enrolled in the SURVIBIO biobank (NCT03518099), including 10 patients with confirmed arterial AMI and 10 controls admitted for non-ischemic acute abdominal pain (Table 1). The final diagnoses among the 10 control patients were mechanical small bowel obstruction (*n* = 6), irritable bowel syndrome (*n* = 3), and renal colic (*n* = 1). Among AMI patients, the median age was 69 years (IQR 60–85), 50% were female, and arterial occlusion was attributed to atherosclerosis in 80% and embolism in 20%. Compared with controls, AMI patients exhibited a marked systemic inflammatory response at presentation, with significantly higher circulating levels of C-reactive protein (*p* < 0.001), serum amyloid A1 (*p* = 0.001), and procalcitonin (*p* = 0.002) (Table 1 and Fig. 1). Leukocyte counts were increased (*p* = 0.01), with trends toward higher neutrophil counts (*p* = 0.02) and MPO levels (*p* = 0.05). Markers of platelet activation and thrombo-inflammation were also elevated, including P-selectin (*p* = 0.02) and plasminogen activator inhibitor-1 (PAI-1; *p* = 0.14). In parallel, fibrinogen γ chain levels were significantly increased (*p* = 0.001), whereas fibrinogen β chain levels and platelet counts did not differ significantly. Fibrinogen α chain, prothrombin, factor XIII, and citrulline levels tended to be lower in AMI patients. The endotoxin levels, markers of bacterial translocation from the intestinal compartment to the vascular compartment, were significantly higher (*p* = 0.025) in AMI patient’s serum (Table 1 and Fig. 1).

**Table 1:** Characteristics of the patient’s cohort

	Control patients *n* = 10 (%)	AMI patients *n* = 10 (%)	*p*-value
Age, years, median (IQR)	37 (27–72)	69 (60–85)	0.01
Female	3 (30)	5 (50)	0.65
Atherosclerosis (vs. arterial embolism)	–	8 (80)	–
Comorbidities			
Tobacco use	3 (30)	3 (30)	1.00
Arterial hypertension	2 (20)	6 (60)	0.17
Dyslipidemia	0 (0)	4 (40)	0.09
Diabetes mellitus	1 (10)	4 (40)	0.30
Myocardial ischemia	0 (0)	2 (20)	0.47
Stroke	0 (0)	2 (20)	0.47
Limb ischemia	0 (0)	2 (20)	0.47
Atrial fibrillation	1 (10)	3 (30)	0.58
Clinical and biological presentation			
Guarding	3 (30)	2 (20)	1.00
Organ failure (SOFA > 2)	3 (30)	5 (50)	0.37
Leukocytes (G/L)	8.4 (5.7–12.6)	15.0 (8.1–22.8)	0.04
Neutrophils (G/L)	5.8 (4.1–8.3)	14.2 (8.4–20.6)	0.02
Lymphocyte (G/L)	1.1 (0.9–1.4)	1.3 (0.8–1.4)	0.73
Platelets (G/L)	233 (179–349)	282 (256–420)	0.10
C-reactive protein (mg/L)	3.0 (1.0–8.2)	52.0 (28.7–154.5)	<0.001
Lactate (mmol/L)	1.6 (1.0–3.6)	1.5 (0.8–2.5)	0.81
Creatinine levels (µmol/L)	71.0 (64.2–92.5)	65.0 (59.5–101.5)	0.61
Follow-up			
ICU admission	0 (0)	5 (50)	0.03
Abdominal surgery	2 (20)	4 (40)	0.63
12-month mortality	0 (0)	2 (20)	0.47

Variables were compared using the Mann–Whitney test. All tests are two-sided.

*IQR* interquartile range, *SOFA* sequential organ failure assessment.

**Fig. 2:**
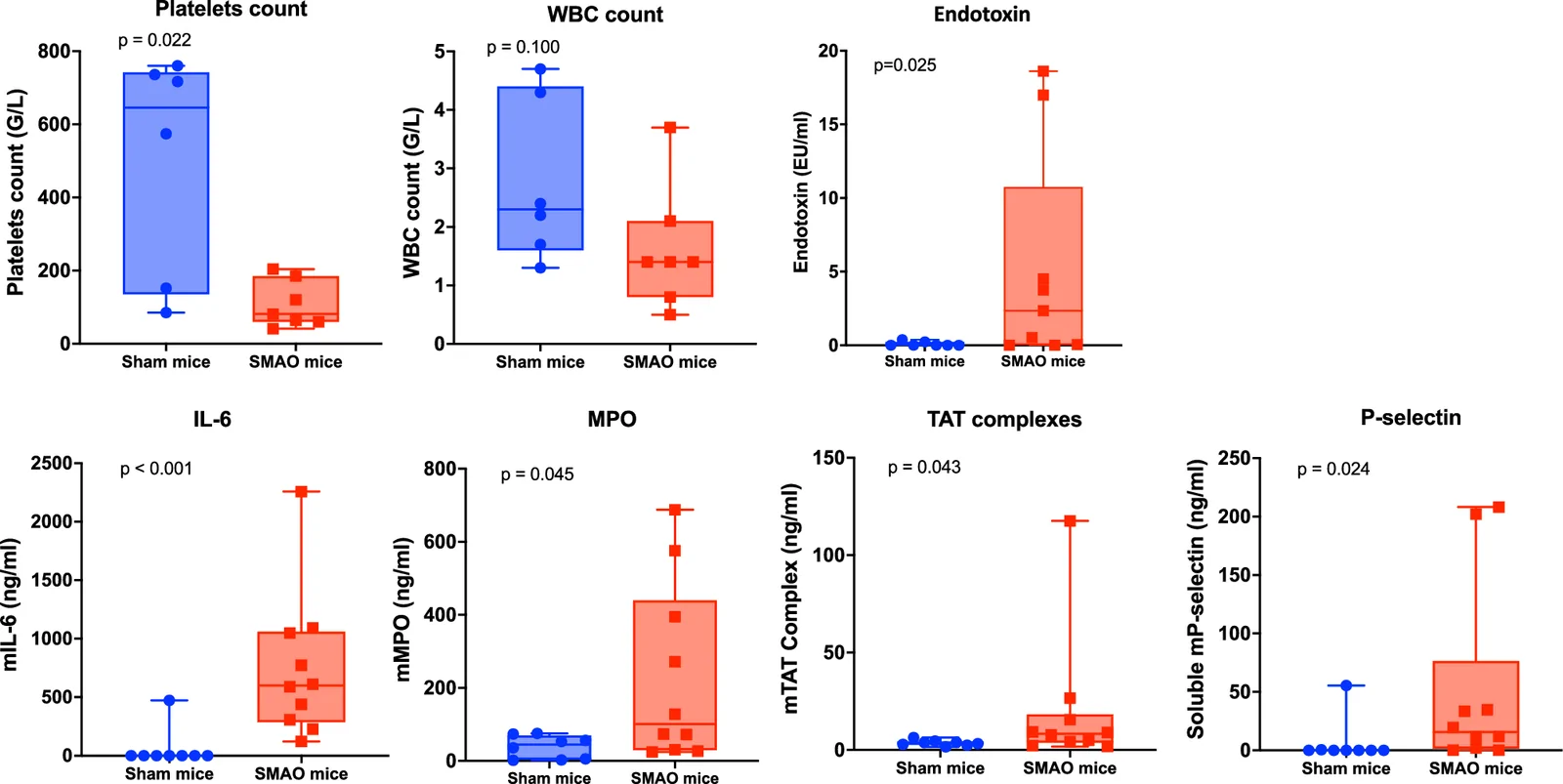
Biomarkers of thrombo-inflammation in mice. Characteristics and inflammation, thrombosis and bacterial markers of control or SMAO mice. Quantitative variables of platelets and white blood cells (WBC) counts are expressed as median and IQR (*n* = 6 Sham mice and *n* = 7 SMAO mice). Variables were compared using the Mann–Whitney test. All tests are two-sided. Plasmatic markers (IL-6, myeloperoxidase (MPO), soluble P-selectin, TAT complexes and endotoxin) are expressed as mean ± SEM (*n* = 8 Sham mice and *n* = 14 SMAO mice). *p* ≤ 0.05 was considered significant. *p*-value is indicated on each graph. *SMAO: superior mesenteric artery occlusion*.

To assess these thrombo-inflammatory alterations under standardized experimental conditions, blood samples were collected in mice subjected to superior mesenteric artery occlusion (SMAO; *n* = 9) or sham surgery (*n* = 7). Complete blood counts were comparable between groups, except for a significant reduction in platelet counts (*p* = 0.02) in SMAO mice. Consistent with the human data, circulating P-selectin (*p* = 0.02) and MPO (*p* = 0.04) levels were increased. In addition, IL-6 levels were markedly elevated (*p* < 0.001), and TAT complexes were increased during IR (*p* = 0.04). Endotoxin levels were significantly higher (*p* = 0.025) in SMAO mice (Fig. 2).

### SMAO disrupts downstream microvascular flow and leads to venular thrombosis after recanalization

To investigate the microvascular origin of thrombo-inflammatory signatures observed in human AMI and in the SMAO model, we used intravital microscopy to visualize mesenteric microcirculatory flow and thrombo-inflammatory events during IR. Preliminary experiments showed that while longer (90-min period) ischemic durations severely compromised animal survival, shorter occlusion times (such as the 30-min period presented in Supplementary Fig. 1) failed to adequately replicate the thrombo-inflammatory features observed in IMA patients. This 1-h model successfully aligned experimental outcomes with our clinical observations (Fig. 7).

**Fig. 3:**
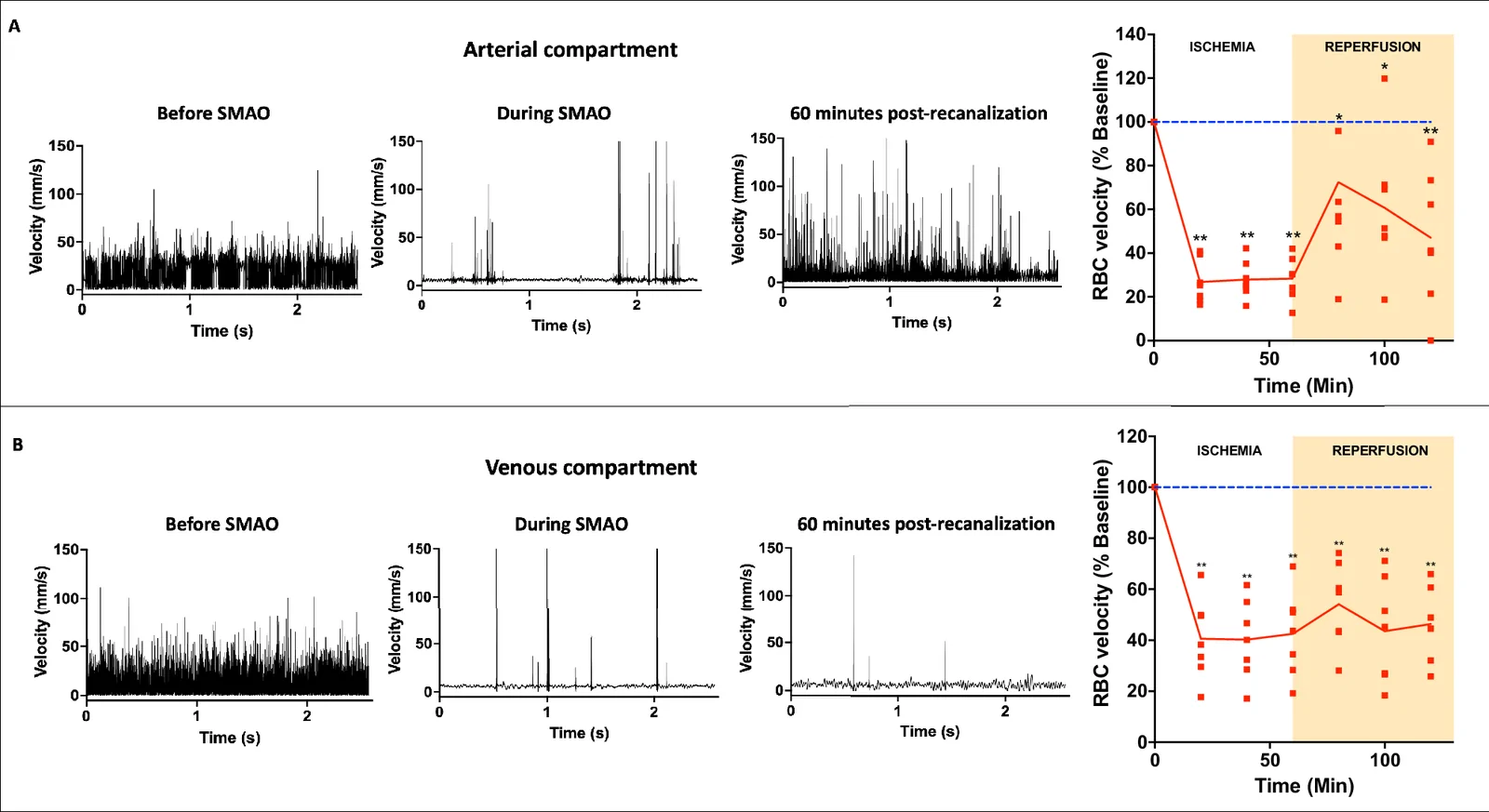
Microvascular blood flow during intestinal ischemia-reperfusion. **A** Arterioles and **B** venules variation in red blood cell velocity at baseline, during superior mesenteric artery (SMA) occlusion (0, 20, 40, and 60 min after SMA occlusion) and after SMA recanalization (20, 40, and 60 min). Results are expressed as percentages relative to baseline values. *n* = 7 mice on, 7 arterioles (50–100 µm diameter) and 7 venules (150–300 µm diameter). Variables were compared using the Mann–Whitney test. All tests are two-sided. Error bars represent SEM. For arterioles: *p* = 0.0012 at 20, 40, 60, 80, and 100 min, and *p* = 0.0022 at 120 min. For venules: *p* = 0.0012 at 20, 40, 60, and 120 min, and *p* = 0.0022 at 80 and 100 min.

**Fig. 4:**
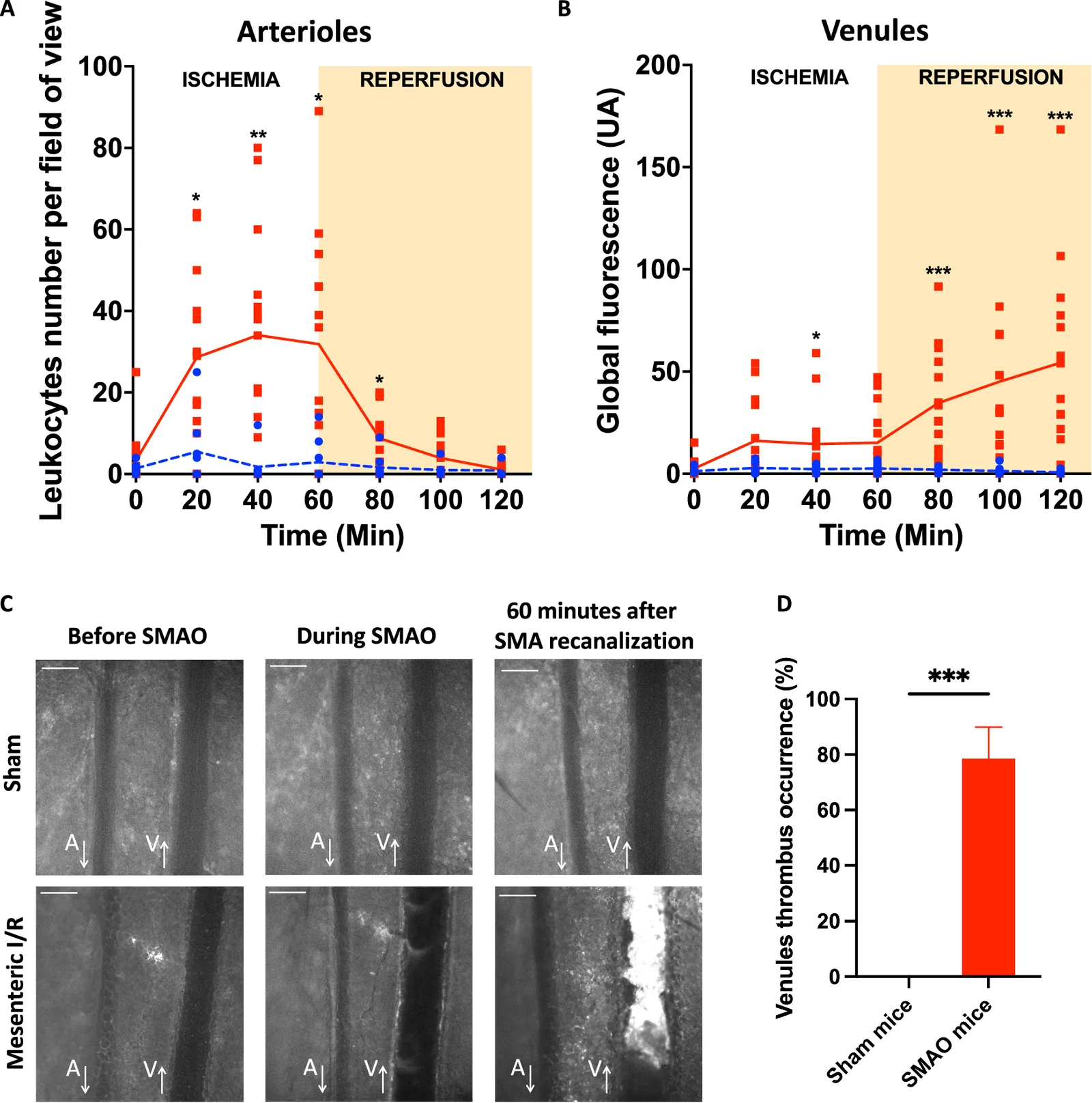
Microvascular alterations following superior mesenteric artery occlusion. **A** Quantification of leukocyte adhesion in mesenteric arterioles before ischemia, during superior mesenteric artery occlusion (SMAO), and after arterial recanalization. Leukocytes were counted per field of view over a 10-s intravital microscopy recording. *p* = 0.008 at 20 min, *p* = 0.001 at 40 min, *p* = 0.005 at 60 and 80 min. **B** Quantification of blood cell stasis in mesenteric venules, expressed as global fluorescence intensity normalized to venular area, measured before ischemia, during SMAO, and after recanalization (three measurements per 10-s recording). *p* = 0.01 at 40 min, *p* = 0.0003 at 80 min, *p* = 0.0004 at 100 min, and *p* = 0.0002 at 120 min. **C** Representative fluorescent intravital microscopy images showing rhodamine 6G–labeled platelets and leukocytes adhering to arteriolar and venular walls downstream of the superior mesenteric artery. Images were acquired before SMAO, immediately before recanalization, and 60 min after recanalization. Arrows indicate blood flow direction. Scale bar, 200 μm. **D** Incidence of venular thrombus formation 60 min after reperfusion, assessed by intravital microscopy (*p* = 0.003). Arterioles analyzed measured 50–100 μm in diameter, and venules 150–300 μm. Variables were compared using the Mann–Whitney test. All tests are two-sided. Data are shown as mean ± SEM. Sham, *n* = 9; SMAO, *n* = 14.

Following superior mesenteric artery occlusion, arteriolar blood flow, assessed by red blood cell velocity, decreased to 27 ± 4% of baseline and partially recovered to 72 ± 19% after 20 min of reperfusion (Fig. 3A). However, arteriolar blood flow progressively declined thereafter, reaching 47 ± 12% at 60 min of reperfusion. Leukocyte rolling and adhesion along arteriolar walls increased during ischemia and progressively resolved during reperfusion, becoming undetectable after 40 min (Fig. 4A, C). In contrast, venular blood flow decreased to 41 ± 6% of baseline during ischemia but remained severely impaired throughout reperfusion (46 ± 6% at 60 min; Fig. 3B). Immediately after reperfusion, venular blood flow resumed but was not sufficient to remove the cell aggregates that had formed during ischemia. These aggregates persisted, gradually enlarged, and formed stable thrombi within 20 min. Thrombi continued to grow throughout the 1-h observation period (Supplementary Videos 1–4), leading to a sustained increase in venular blood cell stasis, as reflected by a 3.6-fold increase in global fluorescence intensity (*p* = 0.007) after 60 min (Fig. 4B, C), and with the formation of venular thrombi in 77% of SMAO mice (Fig. 4D). Thrombi appeared as early as 20 min after reperfusion, progressively enlarged, and remained stable throughout the observation period. No arteriolar thrombi were detected. In the remaining mice, persistent platelet and leukocyte adhesion was observed without overt thrombus formation. No microvascular alterations were observed in sham mice (Fig. 4).

**Fig. 5:**
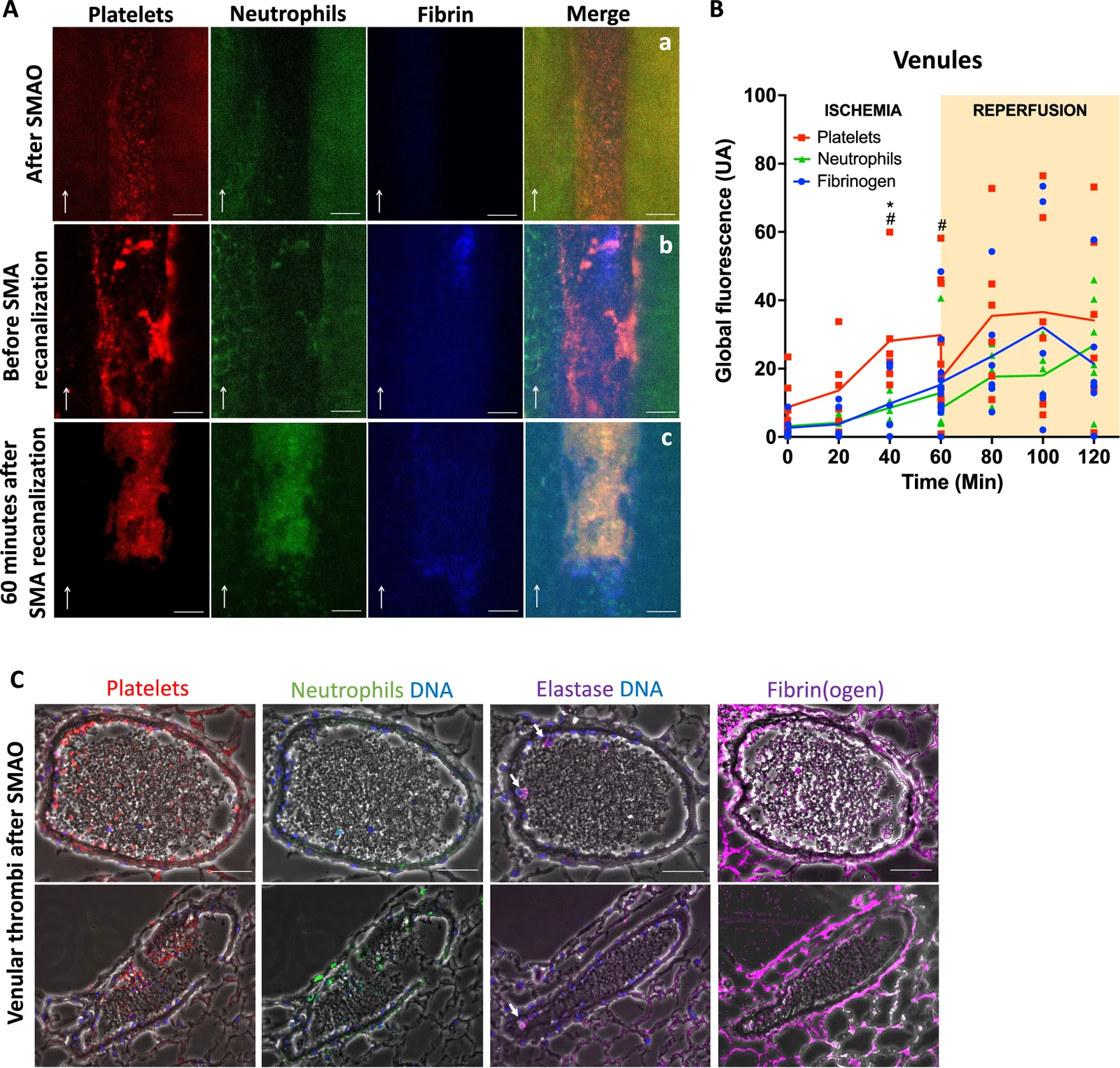
Venular thrombo-inflammatory cell recruitment and thrombus formation. **A** Fluorescent intravital microscopy images showing sequential venular recruitment of platelets (anti-GPIX, red), neutrophils (anti-Ly6G, green), and fibrin deposition (anti-fibrin, blue) downstream of the superior mesenteric artery. Images were acquired immediately after SMAO (a), just before arterial recanalization (b), and 60 min after recanalization (c). Arrows indicate blood flow direction. Scale bar, 50 μm. **B** Venules global fluorescence of specific platelets, neutrophils and fibrin deposition staining, before and during SMAO (0, 20, 40, and 60 min after SMA occlusion) and after SMA recanalization (20, 40, and 60 min). Results are expressed as global fluorescence quantification relative to the venules area observed on a 10 s movie acquisition (three measurements per acquisition). *n* = 7 SMAO mice platelets (squares), neutrophils (triangles) and fibrin deposition staining (circles), venules diameter = 150–300 µm. Error bars represent SEM. Platelets compared with fibrinogen staining: *p = 0.023 at 40 min. Platelets compared with neutrophils staining: #*p* = 0.0022 at 40 min and #*p* = 0.041 at 60 min. **C** Immunofluorescence analysis of venular thrombus sections showing fibrin(ogen) deposition (anti-fibrin(ogen), magenta), neutrophils (anti-Ly6G, green), elastase (anti-neutrophil elastase, magenta), and platelets (anti-CD42b, red). Scale bar, 50 μm.

Fluorescent intravital microscopy and analysis of venular thrombus sections further characterized the cellular dynamics of thrombus formation. Platelet stagnation occurred early after arterial occlusion, whereas neutrophils initially localized predominantly to the perivascular space (Fig. 5A). During ischemia, platelet clusters progressively formed and colocalized first with fibrin and subsequently with neutrophils. Quantification of global fluorescence intensity in the venules confirmed the imaging observations. Platelet fluorescence increased progressively during ischemia, whereas fibrin and neutrophil signals remained low until reperfusion, when both increased markedly. Reperfusion was also associated with a time-dependent accumulation of platelets and neutrophils, together with progressive fibrin deposition (Fig. 5B), consistent with the formation and growth of venular thrombi. Together, these real-time imaging data reveal the dynamic progression of venular thrombus formation during intestinal IR.

Immunofluorescence analysis of retrieved thrombi revealed a distinct architecture, with a platelet- and fibrin-rich core containing entrapped red blood cells, surrounded by an outer layer enriched in fibrin and infiltrated by platelets and neutrophils. The presence of neutrophil extracellular traps (NETs) at the thrombus periphery indicated local neutrophil activation (Fig. 5C).

### Ischemia-reperfusion-induced vascular injury results in intestinal tissue damage

**Fig. 6:**
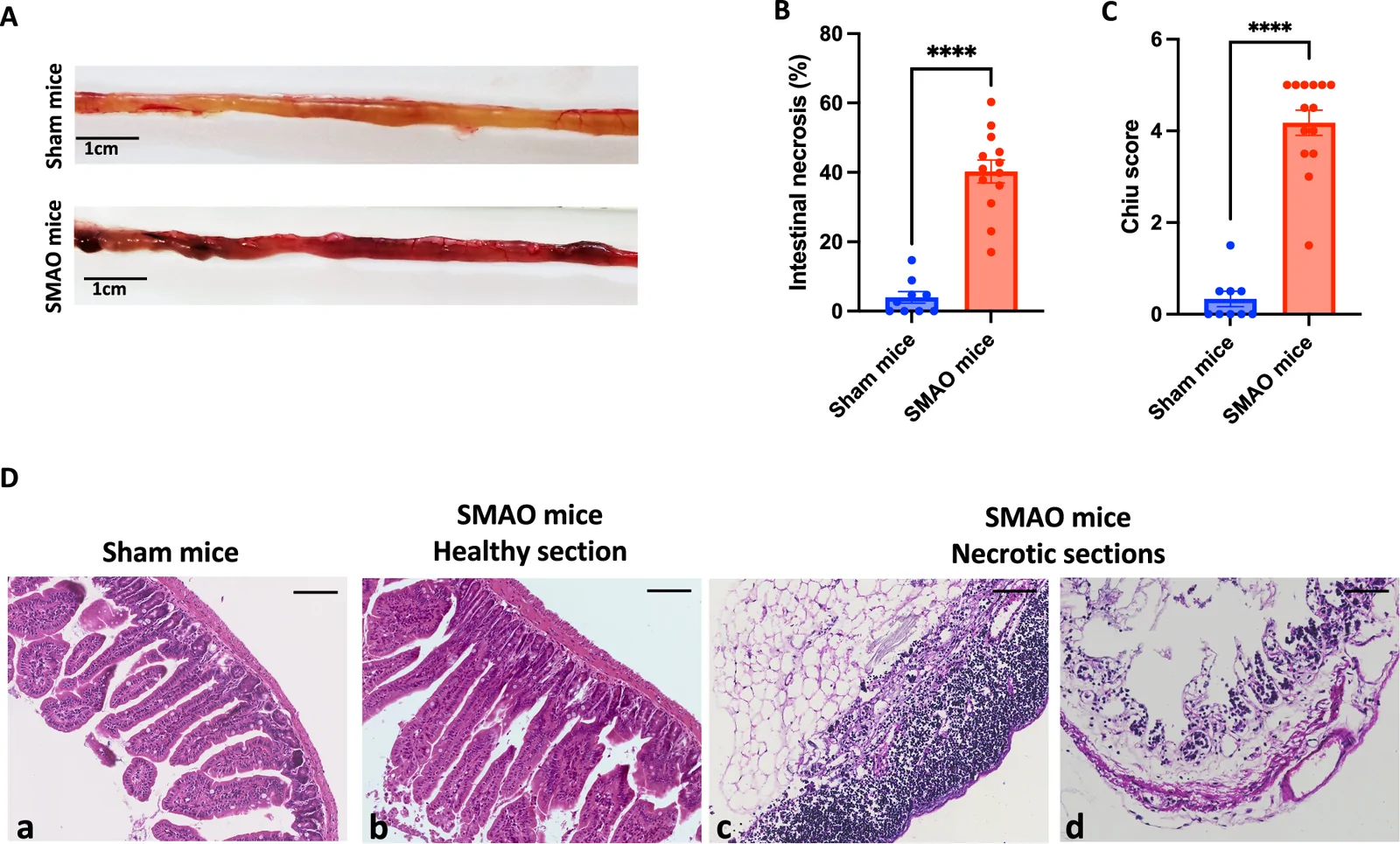
Intestinal injury in experimental and human mesenteric ischemia. **A** Representative macroscopic views of the small intestine from sham mice and mice subjected to superior mesenteric artery occlusion (SMAO; 1 h ischemia followed by 1 h reperfusion), illustrating heterogeneous ischemic and reperfusion-associated lesions. **B** Quantification of the percentage of necrotic small intestine. Sham, *n* = 9; SMAO, *n* = 14. Variables were compared using the Mann–Whitney test. All tests are two-sided (*p* < 0.0001). **C** Histological grading of intestinal injury after ischemia-reperfusion using the Chiu score (0, normal mucosal villi; 5, complete disintegration of the lamina propria with hemorrhage and ulceration). Sham, *n* = 9; SMAO, *n* = 13. Variables were compared using the Mann–Whitney test. All tests are two-sided (*p* < 0.0001). **D** Representative hematoxylin-eosin-stained intestinal sections showing preserved mucosa in sham mice (a), adjacent preserved (b) and necrotic areas (c, d) in SMAO mice. Scale bars, 100 μm. Data are presented as mean ± SEM. Images are representative of 3–4 mice per group.

**Fig. 7:**
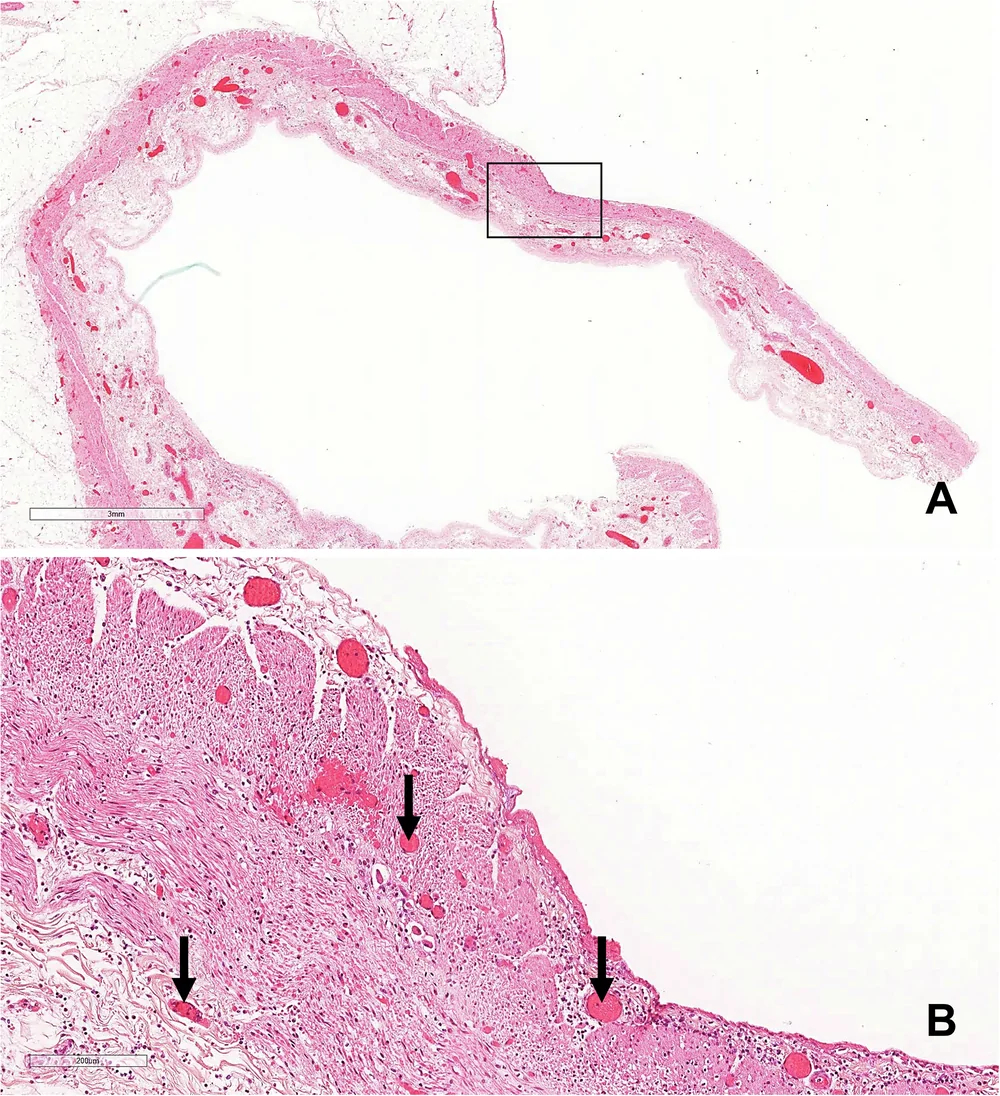
Microvascular thrombosis in human arterial acute mesenteric ischemia. **A** Histological section of infarcted small bowel from a patient with acute mesenteric ischemia caused by atherosclerotic occlusion of the proximal superior mesenteric artery, showing extensive transmural ischemic injury involving all layers of the intestinal wall, including the muscularis externa (hematoxylin and eosin staining; original magnification ×10). **B** Higher magnification of the boxed area in (**A**), focusing on the muscularis externa and revealing myocytic necrosis associated with acute microvascular thrombi within the intestinal wall (arrows) (hematoxylin and eosin staining; original magnification ×200).

Macroscopic examination of the intestine after mesenteric IR revealed heterogeneous ischemic, hemorrhagic, and necrotic lesions along the small bowel (Fig. 6A). Sham mice exhibited preserved macroscopic and microscopic mucosal architecture with intact villi (Fig. 6A, D). Histological analysis of SMAO intestines showed marked spatial heterogeneity, with preserved areas adjacent to severely damaged regions characterized by epithelial lifting, thinning or loss of the lamina propria, hemorrhage, and leukocyte infiltration (Fig. 6D). In SMAO mice, lesions involved 40 ± 3% of the small intestine, with a mean Chiu score of 4, whereas no injury was observed in sham animals (both *p* < 0.001; Fig. 6B, C). Similar histopathological features were observed in resected human specimens from acute mesenteric infarction, where focal microvascular thrombi were occasionally identified (Fig. 7).

## Discussion

In this study, we integrated a deeply phenotyped human AMI biobank with a murine superior mesenteric artery occlusion model to bridge clinical observations with mechanistic in vivo analyses. Although arterial acute mesenteric ischemia is characterized by a systemic thrombo-inflammatory response, circulating biomarkers alone do not identify the microvascular origin of these processes. Using intravital microscopy, we revealed a dissociation between arterial and microvascular reperfusion: while arteriolar flow partially recovers after recanalization, venular perfusion remains profoundly impaired, with early blood cell stasis and stable thrombus formation. This venular-centered thrombo-inflammatory response, initiated during ischemia and amplified during reperfusion, is closely associated with systemic inflammation and may represent a key driver of persistent microvascular dysfunction in AMI.

Our results are consistent with established thrombo-inflammatory paradigms in which platelets act as early responders to ischemic stress, providing a scaffold for fibrin formation and inflammatory cell recruitment^28^. Our detailed, component-specific fluorescence quantification supports this, revealing a strict chronological sequence of platelet, neutrophil recruitment, and fibrin formation. These distinct kinetics offer useful mechanistic clues into the synchronized assembly of venular thrombi during the I/R continuum, laying a foundation for exploring targeted interventional therapies. Neutrophil activation, supported by increased circulating MPO, likely contributes to thrombus maturation and vascular injury^29^. Although NETs were not directly assessed via real-time intravital microscopy, their involvement is demonstrated in our histological thrombus analysis. This is consistent with their known capacity to integrate into fibrin networks, stabilize thrombi, and impair thrombolysis in ischemic conditions^30,31^.

The predominance of venular thrombus formation observed in our model is consistent with the low-shear, stasis-prone environment of post-capillary venules, which favors thrombo-inflammatory amplification, whereas higher arteriolar flow limits stable thrombus formation. Importantly, similar venular-predominant microvascular thrombosis despite upstream arterial recanalization has been described in other ischemic organs, particularly in experimental models of acute ischemic stroke. Using intravital microscopy, Desilles and colleagues demonstrated that microvascular thrombosis following arterial recanalization develops primarily within post-capillary venules, despite restoration of proximal arterial flow^32,33^. Thrombi in these models display a platelet- and red blood cell-rich core stabilized by fibrin, with neutrophils preferentially localized at the thrombus periphery. The observation of comparable venular thrombo-inflammatory events in distinct ischemic vascular beds supports the existence of a shared pathophysiological principle governing microvascular failure after large-vessel reperfusion. This convergence strongly supports the translational relevance of our findings and validates the SMAO model.

Previous studies of intestinal IR have primarily focused on arterial occlusion or on leukocyte rolling as an early inflammatory event. Our findings instead identify the venular compartment as the main site of thrombo-inflammatory injury. Using real-time intravital microscopy, we show that ischemia-induced blood cell stasis persists despite the restoration of blood flow and progressively evolves into stable venular thrombi through the accumulation of platelets, neutrophils, and fibrin. These findings suggest that venular thrombosis is not simply a secondary consequence of IR but a key driver of microvascular dysfunction, highlighting the venular microcirculation as a potential therapeutic target in AMI.

Microvascular thrombo-inflammation translated into substantial tissue injury. Histopathological analysis demonstrated severe and spatially heterogeneous mucosal damage in SMAO mice, quantified using the Chiu scoring system^27^. Comparable features, including focal microvascular thrombi, were observed in resected human intestinal specimens, supporting the clinical relevance of microvascular involvement in AMI. In humans, however, direct visualization of venular microthrombi is not feasible, and histological analyses are restricted to resected specimens, in which advanced ischemic necrosis often precludes precise characterization of the microvascular compartment involved.

Our results extend previous experimental work showing that restoration of arterial patency does not guarantee sustained microvascular reperfusion. Using laser Doppler flowmetry in a rat ischemia-reperfusion model, Aksoy et al. showed that capillary flow recovers after short ischemic durations but deteriorates following prolonged ischemia despite arterial recanalization^34^. Our intravital microscopy data refine these observations by identifying the venular compartment as a critical site of persistent flow impairment characterized by intense by thrombo-inflammatory activity. Blood cell stasis was directly observed during live imaging. During ischemia, trapped fluorescent cells moved passively back and forth with the arterial pulse without showing stable adhesion. Because individual cells could no longer be reliably identified after reperfusion, when thrombi progressively formed, we used global fluorescence intensity within venular segments as a surrogate measure of blood cell stasis and thrombus formation. Our imaging was focused on mesenteric arterioles and venules and did not evaluate capillary perfusion. Nevertheless, venular occlusion is likely to reduce downstream capillary blood flow and thereby contribute to the intestinal lesions observed after reperfusion.

Beyond microvascular thrombosis, the intestine displays unique features that may amplify thrombo-inflammation compared with other ischemic organs. Unlike the brain or myocardium, the gut is not a sterile organ and harbors a dense immune cell compartment and complex microbiota. Systemic inflammatory response syndrome (SIRS) is reported in 25–100% of AMI cases^35,36^, whereas ischemic injury involving the heart or brain is associated with SIRS in only 3–22% of cases^37–39^. Intestinal ischemia rapidly induces microbiota alterations, bacterial overgrowth, and translocation of anaerobic species that may escape detection by standard blood cultures (“occult bacteremia”)^40^. Bacterial products and endotoxins further amplify systemic inflammation and may aggravate ischemic injury through impaired epithelial renewal^41^, increased epithelial apoptosis^42^, and microvascular steal phenomena mediated by nitric oxide synthase activation^43^. In line with this concept, experimental models have shown reduced microbial translocation, SIRS, and mortality with oral antibiotics or in germ-free animals^44,45^. Consistently, clinical data support a protective effect of antibiotic strategies in AMI^25,44,45^, motivating the ongoing randomized ORIAMI trial (ORal antibiotics in Acute Mesenteric Ischemia, NCT06387147).

These results may also help reinterpret clinical observations linking vascular comorbidities to worse outcomes. Diabetes has been associated with increased severity of AMI due to mesenteric venular thrombosis^46^, suggesting that pre-existing microvascular disease may amplify venular thrombo-inflammation during ischemia-reperfusion. In arterial AMI, such mechanisms may contribute to the persistently high mortality, which remains in the range of 20–30% even when patients are diagnosed early, managed in expert centers, and treated with prompt arterial revascularization^47^. In this context, the present data provide a biological rationale supporting the current empirical use of anticoagulation in AMI management, while underscoring the limitations of arterial recanalization alone.

Our validated mouse model provides a translational platform to evaluate existing therapeutic strategies. Future studies will determine how these treatments influence venular thrombo-inflammation during acute IR injury. Importantly, our findings provide a foundation for future pharmacological and interventional studies targeting thrombo-inflammatory pathways, not only in arterial AMI but also in other IR settings, including non-occlusive mesenteric ischemia (NOMI) and low-flow states encountered in critical care or during major cardiovascular surgery. A better understanding of these downstream mechanisms may also facilitate the identification of novel diagnostic and prognostic biomarkers, another major unmet need in AMI^48^.

This study has several limitations. First, the clinical cohort was limited to a single center and included a relatively small number of patients, which precluded robust analyses according to disease severity. In addition, serum samples were collected only at admission to preserve routine clinical management and did not allow longitudinal assessment after reperfusion. Nevertheless, these rare samples provide a unique snapshot of the thrombo-inflammatory response at the time of AMI diagnosis, and the biomarker profile was remarkably consistent across patients and closely mirrored our experimental findings. Second, although our study combines clinical observations with real-time intravital imaging in a preclinical mouse model, additional studies targeting specific thrombo-inflammatory pathways will be required to establish their causal contribution to venular thrombosis. Finally, the 1-h ischemia protocol was selected as the best compromise between reproducible thrombo-inflammatory injury and animal survival, based on model optimization experiments. Despite these limitations, our study provides new mechanistic insight into the development of venular thrombo-inflammation during AMI and establishes a translational platform for future therapeutic studies. In conclusion, mesenteric ischemia-reperfusion induces a venular-centered thrombo-inflammatory response that persists despite arterial recanalization and may contribute to sustained microvascular dysfunction and intestinal injury. By integrating human biomarker profiling with mechanistic intravital imaging, this study provides a unifying framework linking systemic inflammation, microvascular thrombosis, and poor outcomes in AMI and supports targeting thrombo-inflammation as a therapeutic complement to arterial revascularization.

## Supplementary information


Supplementary Information
Description of Additional Supplementary Files
Supplementary Data 1
Supplementary Data 2
Supplementary Data 3
Supplementary Data 4
Supplementary Data 5
Supplementary_video_1
Supplementary_video_2
Supplementary_video_3
Supplementary_video_4


## Data Availability

The datasets generated and analyzed during the current study comprise both human clinical cohort data and experimental preclinical datasets. Human clinical data associated with Table 1 and Fig. 1 originate from protocols conducted in accordance with the Declaration of Helsinki and Good Clinical Practice guidelines under ClinicalTrials.gov identifier NCT03518099. To protect participant confidentiality and maintain compliance with European General Data Protection Regulation (GDPR) standards, human clinical datasets are under controlled access. De-identified participant data are available from the corresponding author upon reasonable request, subject to formal evaluation and approval by the SURVI/TSUNAMI steering committee and execution of a data-use agreement. Access requests will be evaluated and responded to within 30 business days. The data-use agreement stipulates that data be used strictly for non-commercial academic research relevant to vascular intestinal diseases, housed on secure servers, and without attempt to re-identify or redistribute individual participant data. For the remaining figures, experimental and preclinical datasets are not subject to human clinical regulatory restrictions. No datasets requiring deposition in public repositories were generated during this study, and therefore, no accession codes are applicable. All relevant Source data underlying the findings are included in the Supplementary Source data file or available upon reasonable request. The Source data for Fig. 2 is in Supplementary Data 1. The Source data for Fig. 3 is in Supplementary Data 2. The Source data for Fig. 4 is in Supplementary Data 3. The Source data for Fig. 5 is in Supplementary Data 4. The Source data for Fig. 6 is in Supplementary Data 5.
