# Supplementary material for: Venular-centered thrombo-inflammation drives microvascular failure after arterial recanalization in acute mesenteric ischemia
Source: Commun Med (Lond). 2026 Aug 28;6:472. doi: 10.1038/s43856-026-01878-y (PMC13530138; doi:10.1038/s43856-026-01878-y)
Supplement: Supplementary file 1 [file 43856_2026_1878_MOESM1_ESM.pdf]

Supplementary Figure 1

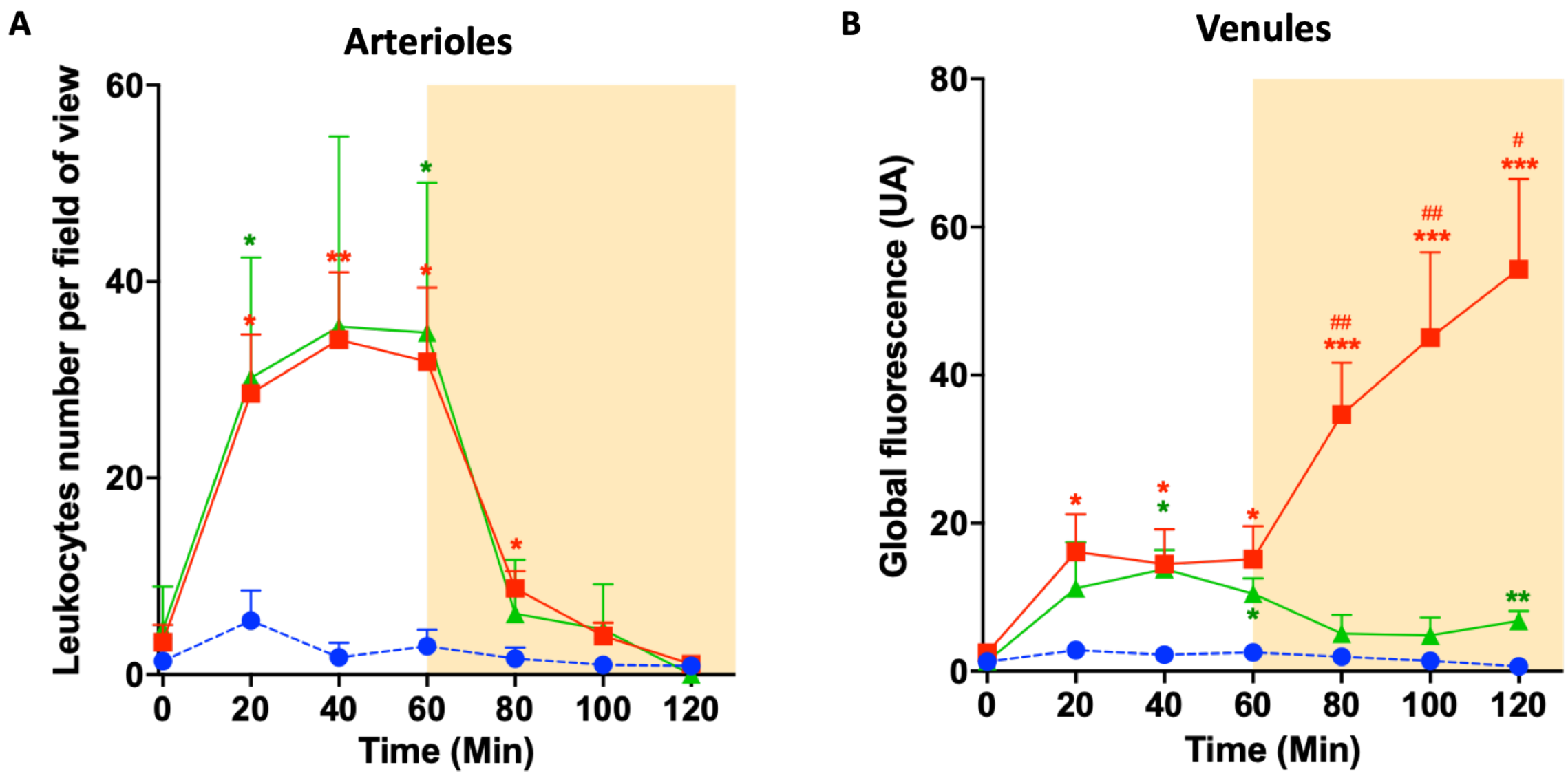

Kinetics of microvascular damages at 30 & 60 minutes post-SMAO.
(A) Arterioles leukocytes count and **(B)** Venules global fluorescence, before and during SMAO (0, 20, 40 & 60 minutes after SMA occlusion) and after SMA recanalization (20, 40 & 60 minutes). Results are expressed as leukocytes number observed per field of view, on 10 seconds movie acquisition. Sham mice (circles, n=9), 30-minutes SMAO mice (triangles, n=5) and 60minutes-SMAO mice (squares, n=14), arterioles diameter = 50 to 100µm and venules diameter = 150 to 300µm. Variables were compared using the Mann–Whiney test. All tests are two-sided. Error bars represent SEM. *P<0.05, **P<0.01 and ***P<0.001, compared with sham; #P<0.05 and ##P<0.01, compared with 30 minutes-SMAO.

Supplementary Methods

Proteomic serum analysis (untargeted LC–MS/MS)

Serum proteins were first depleted of the 12 most abundant plasma proteins using the Proteome Purify 12 Human kit (R&D Systems) and subsequently concentrated with Amicon Ultra centrifugal filters (Merck Millipore). For each sample, 10 µg of total protein were adjusted to 120 µL of denaturation buffer (4 M urea, 1.5 M thiourea, 50 mM Tris–HCl pH 8.3). Proteins were reduced with 10 mM dithiothreitol for 30 minutes at room temperature and alkylated with 55 mM iodoacetamide for 20 minutes. Samples were digested sequentially, first with 500 ng Lys-C for 3 hours at room temperature, then, after threefold dilution in Milli-Q water, with 500 ng trypsin (Promega) for 16 hours at room temperature. Digestion was terminated by adding formic acid to a final concentration of 3%, and peptide mixtures were stored at –20 °C until analysis.

Peptides were desalted with ZipTip µ-C18 tips (Millipore) before LC–MS/MS analysis on a Q-Exactive Plus mass spectrometer coupled to a Nano-LC Proxeon 1000 system and Easy-Spray ion source (Thermo Scientific). Chromatographic separation was performed using a PepMap C18 precolumn (2 cm, 75 µm i.d., 3 µm) and a Pepmap-RSLC analytical column (50 cm, 75 µm i.d., 2 µm) under a 98-minute gradient from 5% to 35% acetonitrile (0.1% formic acid) at 300 nL/min, for a total run time of 120 minutes. Full MS scans were acquired at 70,000 resolution (m/z 375–1500; AGC 3×10^6), and HCD MS/MS spectra were recorded in Top20 data-dependent mode at 17,500 resolution (AGC 2×10^5; NCE 30%; isolation window 1.4 Da; dynamic exclusion 30 s). Maximum injection times were 50 ms for MS and 45 ms for MS/MS.

Raw data were processed using Proteome Discoverer 2.2 with Mascot 2.5.1. Searches were performed with tolerances of 6 ppm for precursor ions and 0.02 Da for fragments, allowing up to two missed cleavages and considering oxidation (Met), phosphorylation (Ser/Thr/Tyr), carbamidomethylation (Cys), and N-terminal acetylation as variable modifications. Peptide-spectrum matches were validated at a 1% false discovery rate (FDR) using Percolator. Label-free quantification was performed with Progenesis QI 3.0 through chromatogram alignment, normalization on total peptide abundance, statistical analysis of peptide features, and subsequent peptide–protein identification. Protein quantification followed the Hi-3 approach, and only proteins with at least two unique peptides were considered. Differentially abundant proteins were defined by a p-value < 0.05
