# Supplementary material for: Venular-centered thrombo-inflammation drives microvascular failure after arterial recanalization in acute mesenteric ischemia
Source: Commun Med (Lond). 2026 Aug 28;6:472. doi: 10.1038/s43856-026-01878-y (PMC13530138; doi:10.1038/s43856-026-01878-y)
Supplement: Supplementary file 2 [file 43856_2026_1878_MOESM3_ESM.pdf]

Figure 2 - Cell counts & dosages

Identification number	SMAO1 CTL0	Platelets (G/L)	Leukocytes (G/L)	Endotoxin (EU/mL)	IL6 (ng/mL)	MPO (ng/mL)	TAT (ng/mL)	P-Selectin (ng/mL)
1	0	152,0	2,4	0,0	0,0	36,1	4,6	0,0
2	0	736,0	2,2	0,0	0,0	5,8	6,4	0,0
3	0			0,0	0,0	73,7	3,3	0,0
4	0	85,0	1,3	0,2	0,0	2,8	1,6	0,0
5	0	717,0	4,7	0,0	0,0	53,2	3,9	55,5
6	0	760,0	1,7	0,0	472,9	56,6	2,5	0,0
7	0	574,0	4,3	0,4	0,0	75,2	4,0	0,0
8	0				0,0	1,7	2,9	0,6
9	1	60,0	1,4	4,5	773,9	73,8	117,6	11,7
10	1	81,0	1,4	0,5	1048,7	128,1	9,3	33,4
11	1	185,0	1,4	3,8	439,7	24,8	1,8	2,0
12	1	41,0	0,5	0,0	306,8	30,6	15,6	0,0
13	1			2,3	1092,5	27,1	4,3	34,6
14	1	64,0	2,1	0,0	2257,4	575,6	9,0	208,0
15	1	120,0	0,8	0,0	589,3	394,5	26,6	11,7
16	1	204,0	3,7	17,0	122,2	687,4	7,7	201,9
17	1			18,6	226,6	72,3	2,2	19,5
18	1				611,1	271,6	5,1	0,0
