# Supplementary material for: Venular-centered thrombo-inflammation drives microvascular failure after arterial recanalization in acute mesenteric ischemia
Source: Commun Med (Lond). 2026 Aug 28;6:472. doi: 10.1038/s43856-026-01878-y (PMC13530138; doi:10.1038/s43856-026-01878-y)
Supplement: Supplementary file 3 [file 43856_2026_1878_MOESM4_ESM.pdf]

Figure 3 - Doppler analysis

Experimentation time (min)	SMAO mice - Venules (Figure 3B)							SMAO mice - Arterioles (Figure 3A)						
0	100	100	100	100	100	100	100	100	100	100	100	100	100	100
20	29,58	65,58	33,42	17,69	38,29	49,59	49,92	29,58	65,58	33,42	17,69	38,29	49,59	49,92
40	28,53	61,64	32,36	17,15	40,17	46,67	54,98	28,53	61,64	32,36	17,15	40,17	46,67	54,98
60	28,34	68,92	34,47	19,18	43,71	51,99	50,93	28,34	68,92	34,47	19,18	43,71	51,99	50,93
80	28,1	70,33	43,63	74,19	43,25	58,81	60,5	28,1	70,33	43,63	74,19	43,25	58,81	60,5
100	26,54	71,16	27	18,34	45,26	51,5	65,1	26,54	71,16	27	18,34	45,26	51,5	65,1
120		65,94	25,84	32,09	44,56	48,89	60,72		65,94	25,84	32,09	44,56	48,89	60,72
