# Supplementary material for: Venular-centered thrombo-inflammation drives microvascular failure after arterial recanalization in acute mesenteric ischemia
Source: Commun Med (Lond). 2026 Aug 28;6:472. doi: 10.1038/s43856-026-01878-y (PMC13530138; doi:10.1038/s43856-026-01878-y)
Supplement: Supplementary file 4 [file 43856_2026_1878_MOESM5_ESM.pdf]

Figure 4B - Venules - Global fluorescence (UA)

Experimentation time (min)	Sham									Mesenteric I/R													
0		1,58	0,85	0,34	0,81	0,92	0,88	4,36	0,61	0,06	0,65	0,24	1,11	0,94	1,04	0,11	0,33	2,53	5,95	5,50	1,01	0,27	15,24
20		0,17	0,45	0,05	1,78	1,69	5,59	5,45	7,45	1,05	1,58	4,60	2,80	1,06	2,03	33,68	2,41	49,83	36,33	17,42	7,20	54,07	11,61
40		0,02	1,34	0,04	3,64	0,41	3,01	4,92	4,51	0,47	0,64	6,44	5,83	2,43	0,95	59,04	13,35	46,57	17,00	7,18	8,42	20,60	13,79
60	0,86	0,03	0,75	0,07	3,93	5,19	6,82	1,95	3,26	0,00	5,25	0,20	1,93	0,91	0,80	43,10	11,58	47,20	24,93	19,84	10,50	36,91	8,94
80		0,05	0,83		3,72	0,34	3,26	3,76	1,69	0,34	91,58	8,66	12,11	25,79	6,04	54,83	34,91	61,52	19,43	25,67	33,47	47,16	63,88
100		0,02	0,23	0,01	1,66	0,21	0,17	6,43	2,46	0,00	168,51	31,88	13,85	81,78	7,95	48,17	19,10	67,91	30,58	14,42	29,75	48,15	68,40
120	0,49	0,13	0,55	0,03	0,55	0,92	0,34	2,57	0,19	0,01	168,51	21,87	16,91	77,46	4,41	54,02	36,59	86,15	29,17	28,91	57,71	71,73	106,56

Figure 4A - Arterioles - Leukocytes count (per field of view)

Experimentation time (min)	Sham									Mesenteric I/R													
0		2	1	2	0	2	0	0	4	7	1	2	25	0	1	0	0	0	0	5	0	0	5
20		10	4	0	0	25	0	0	5	50	13	10	38	30	29	18	64	63	0	17	40	0	
40		12	1	0	0	0	0	0	1	60	21	9	34	44	38	14	77	80	0	20	41	0	39
60	8	4	0	0	0	14	0	0	0	0	18	15	46	39	36	12	54	89	0	59	46	0	
80		9	1	0	0	0	0	3	0	12	19	9	20	3	12	0	10	6		3	10	0	10
100		5	0	1	0	0	0	1	1	10	13	5	11	0	7	0	4	0		0	0	0	1
120	4	4	0	0	0	0	0	0	0	2	6	1	1	1	3	0	0	0		0	0	0	0

Figure 4D - Venules thrombus occurrence

Sham mice	SMAO mice Thrombus1 NoThrombus0
0	1
0	1
0	1
0	0
0	1
0	0
0	1
0	1
0	1
	1
	0
	1
	1
	1
