# Supplementary material for: Venular-centered thrombo-inflammation drives microvascular failure after arterial recanalization in acute mesenteric ischemia
Source: Commun Med (Lond). 2026 Aug 28;6:472. doi: 10.1038/s43856-026-01878-y (PMC13530138; doi:10.1038/s43856-026-01878-y)
Supplement: Supplementary file 5 [file 43856_2026_1878_MOESM6_ESM.pdf]

Figure 5B - Triple staining - Intravital microscopy - Global fluorescence (UA)

Experimentation time (min)	Fibrinogen							Platelets							Neutrophils						
0	0,45	0,78	0,21	0,79	1,08	0,43	0,94	4,87	0,56	3,05	0,43	1,96	2,12	0,47	0,21	3,75	5,16	2,05	1,33	1,36	0,27
0,1	8,80	1,60	0,45	1,28	3,60	0,43	0,85	14,34	1,53	3,11	7,81	23,42	2,12	5,28	3,35	3,29	4,08	2,05	4,68	1,36	7,44
20	8,80	1,15	0,92	0,31	0,15	11,07	0,85	18,27	8,21	4,69	15,12	1,44	33,75	5,28	3,96	4,85	1,26	6,98	1,77	6,15	7,44
40	21,50	3,66	20,47	3,54	0,15	9,26	0,14	59,94	15,24	28,71	24,37	18,51	22,07	3,05	9,71	4,50	13,68	10,38	7,73	5,05	4,71
60	48,40	14,44	7,18	8,84	0,09	12,70	0,32	58,18	46,01	14,28	21,34	27,68	11,40	18,45	10,46	4,59	13,16	40,63	4,51	4,17	22,54
60,1	28,64	18,87	8,12	14,13	16,60	9,36	0,58	10,59	17,59	0,89	28,29	0,42	44,97	5,95	10,78	8,42	7,28	15,70	4,41	3,95	1,53
80	54,26	29,85	7,28	20,94	14,19	15,31	5,03	72,73	17,82	10,91	44,78	38,55	27,80	14,57	19,54	15,48	11,19	27,18	23,86	8,71	3,35
100	73,39	24,50	2,08	68,88	11,41	12,40	10,45	76,46	9,59	6,47	64,20	28,92	33,71	9,79	12,97	11,44	19,94	22,44	10,85	30,20	22,21
120	57,72	12,92	0,21	26,33	15,98	14,91	3,05	73,15	14,31	1,25	56,96	23,08	35,85	10,40	18,85	40,29	3,76	30,69	21,10	45,97	19,04
