# Supplementary material for: Venular-centered thrombo-inflammation drives microvascular failure after arterial recanalization in acute mesenteric ischemia
Source: Commun Med (Lond). 2026 Aug 28;6:472. doi: 10.1038/s43856-026-01878-y (PMC13530138; doi:10.1038/s43856-026-01878-y)
Supplement: Supplementary file 6 [file 43856_2026_1878_MOESM7_ESM.pdf]

Figure 6C - Anathomopathology - Chiu Score

Sham mice									SMAO mice														
0	1,5	0,5	0,5	0	0,5	0	0	0	4,5	5	5	5	5	5	3,5	4	3	3,5		4,5	5	4	1,5

Figure 6B - Anathomopathology - % Lesions

Sham mice									SMAO mice														
4,7	2,7	0	0	0	15	0	4,7	8,9	43	60	50	36		31	40	17	54	45		41	46	23	38
