# Supplementary material for: Venular-centered thrombo-inflammation drives microvascular failure after arterial recanalization in acute mesenteric ischemia
Source: Commun Med (Lond). 2026 Aug 28;6:472. doi: 10.1038/s43856-026-01878-y (PMC13530138; doi:10.1038/s43856-026-01878-y)
Supplement: Supplementary file 7 [file 43856_2026_1878_MOESM2_ESM.docx]

**Description of Additional Supplementary Files**

**Supplementary data**

**Supplementary Data 1:** Source data underlying the graphs in Figure 2 - Biomarkers of thrombo-inflammation in mice.

**Supplementary Data 2:** Source data underlying the graphs in Figure 3 - Microvascular blood flow during intestinal ischemia-reperfusion.

**Supplementary Data 3:** Source data underlying the graphs in Figure 4 - Microvascular alterations following superior mesenteric artery occlusion.

**Supplementary Data 4:** Source data underlying the graph in Figure 5 - Venular thrombo-inflammatory cell recruitment and thrombus formation.

**Supplementary Data 5:** Source data underlying the graphs in Figure 6 - Intestinal injury in experimental and human mesenteric ischemia.

**Supplementary videos**

**Supplementary_video_1:** Kinetics of microvascular damages post-SMAO in the arterioles and venules of sham mice during ischemic phase. Fluorescent intravital microscopy videos illustrating the marked adhesion of rhodamine 6G-labelled platelets and leukocytes in venules, downstream of the superior mesenteric artery (arrows indicate blood flow direction). Labelled platelets and leukocytes are observed during ischemic and reperfusion phase. Bar = 100µm.

**Supplementary_video_2:** Kinetics of microvascular damages post-SMAO in the arterioles and venules of sham mice during reperfusion phase. Fluorescent intravital microscopy videos illustrating the marked adhesion of rhodamine 6G-labelled platelets and leukocytes in venules, downstream of the superior mesenteric artery (arrows indicate blood flow direction). Labelled platelets and leukocytes are observed during ischemic and reperfusion phase. Bar = 100µm.

**Supplementary_video_3:** Kinetics of microvascular damages post-SMAO in the arterioles and venules of SMAO mice during ischemic phase. Fluorescent intravital microscopy videos illustrating the marked adhesion of rhodamine 6G-labelled platelets and leukocytes in venules, downstream of the superior mesenteric artery (arrows indicate blood flow direction). Labelled platelets and leukocytes are observed during ischemic and reperfusion phase. Bar = 100µm.

**Supplementary_video_4:** Kinetics of microvascular damages post-SMAO in the arterioles and venules of SMAO mice during reperfusion phase. Fluorescent intravital microscopy videos illustrating the marked adhesion of rhodamine 6G-labelled platelets and leukocytes in venules, downstream of the superior mesenteric artery (arrows indicate blood flow direction). Labelled platelets and leukocytes are observed during ischemic and reperfusion phase. Bar = 100µm.
